# Genome-wide identification and structural analysis of the *BMP* gene family in *Triplophysa dalaica*

**DOI:** 10.1186/s12864-024-10049-z

**Published:** 2024-02-19

**Authors:** Yizheng Zhang, Jinhui Yu, Rui Han, Zhigang Ma, Meng Zhang, Yikai Li, Yongtao Tang, Guoxing Nie, Chuanjiang Zhou

**Affiliations:** 1 College of Fisheries, Engineering Technology Research Center of Henan Province for Aquatic Animal Cultivation, Henan Normal University, Xinxiang, 453007 People’s Republic of China; 2 College of Life Sciences, Henan Normal University, Xinxiang, 453007 People’s Republic of China

**Keywords:** *Triplophysa dalaica*, Bone morphogenetic protein, Gene family, Gene expression

## Abstract

**Background:**

Bone morphogenetic proteins *(BMPs*) are part of the transforming growth factor beta (TGF-β) superfamily and play crucial roles in bone development, as well as in the formation and maintenance of various organs. *Triplophysa dalaica*, a small loach fish that primarily inhabits relatively high elevations and cooler water bodies, was the focus of this study. Understanding the function of *BMP* genes during the morphogenesis of *T. dalaica* helps to clarify the mechanisms of its evolution and serves as a reference for the study of *BMP* genes in other bony fishes. The data for the *T. dalaica* transcriptome and genome used in this investigation were derived from the outcomes of our laboratory sequencing.

**Results:**

This study identified a total of 26 *BMP* genes, all of which, except for *BMP1*, possess similar TGF-β structural domains. We conducted an analysis of these 26 *BMP* genes, examining their physicochemical properties, subcellular localization, phylogenetic relationships, covariance within and among species, chromosomal localization, gene structure, conserved motifs, conserved structural domains, and expression patterns. Our findings indicated that three *BMP* genes were associated with unstable proteins, while 11 *BMP* genes were located within the extracellular matrix. Furthermore, some *BMP* genes were duplicated, with the majority being enriched in the GO:0008083 pathway, which is related to growth factor activity. It was hypothesized that genes within the *BMP1/3/11/15* subgroup (Group I) play a significant role in the growth and development of *T. dalaica*. By analyzing the expression patterns of proteins in nine tissues (gonad, kidney, gill, spleen, brain, liver, fin, heart, and muscle), we found that *BMP* genes play diverse regulatory roles during different stages of growth and development and exhibit characteristics of division of labor.

**Conclusions:**

This study contributes to a deeper understanding of *BMP* gene family member expression patterns in high-altitude, high-salinity environments and provides valuable insights for future research on the *BMP* gene family in bony fishes.

**Supplementary Information:**

The online version contains supplementary material available at 10.1186/s12864-024-10049-z.

## Introduction

Bone morphogenetic proteins (*BMPs*) constitute a highly potent class of growth factors within the larger TGF-β superfamily. The biological actions of *BMPs* were initially described in the 1960s when they were found to stimulate the formation of ectopic bone [1]. Since the successful cloning and identification of the first *BMP* genes in the 1980s [2], more than 40 *BMP* genes have been identified. *BMPs* are crucial for osteoblast differentiation and bone formation and have been implicated in cancer development by promoting the growth and invasion of cancer cells [3, 4]. These genes also regulate various physiological systems, including the circulatory, gastrointestinal, pulmonary, urinary, reproductive, and neurological systems [5], playing critical roles in embryonic development, growth, and differentiation [6]. Based on sequence homology, *BMP* family genes are divided into groups according to their roles in cell differentiation and growth: *BMP1/3/11/15* (Group I), *BMP12/13/14* (Group II), *BMP2/4/16* (Group III), *BMP9/10* (Group IV), and *BMP5/6/7/8* (Group V) [7, 8]. *BMP1* encodes a bone morphogenetic protein, which is a metalloproteinase responsible for regulating the deposition of extracellular matrix in vertebrate fibers. It also plays a vital role in various extracellular matrix metabolism and regulatory processes, contributing to essential biological functions [9]. *BMP2* and *BMP4* have well-established roles in critical processes such as embryonic development and the creation and differentiation of adipose precursor cells [10]. *BMP4* influences the differentiation and proliferation of lipogenic progenitor cells, with differentiated adipocytes secreting *BMP4* [11]. *BMP9* is involved in chronic liver disease (CLD), where its elevated expression promotes fibrosis in fibrotic livers [12]. *BMP7* promotes the neuronal differentiation of bone marrow mesenchymal stem cells (BMSCs), while *BMP8* plays a crucial role in preventing premature luteinization of granulosa cells for ovarian follicle development [13, 14]. *BMP* genes exhibit variable expression in various carp tissues, including the gills, gut, liver, spleen, skin, heart, gonads, muscle, kidneys, cephalic kidneys, brain, and blood [8].

*T. dalaica* is found primarily in northern China in the Yellow River tributaries and in artesian water basins such as Inner Mongolia's Dali Lake [15]. *T. dalaica* thrives in high-altitude environments characterized by low water temperatures, especially in high-salt alkaline waters such as still lakes and river slopes. This species exhibits remarkable adaptations to plateau conditions, including its ability to acclimate to low-oxygen aquatic habitats and endure cold temperatures [15]. This harsh environment requires specific physiological adaptations for survival and reproduction, and the *BMP* gene family is involved in many physiological and metabolic processes, including bone and cartilage formation. Therefore, investigating the role of *BMP* genes in the adaptation of this species to extreme conditions will help to unravel the underlying mechanisms of its molecular adaptation and evolution and provide some assistance for subsequent studies of the *BMP* gene family in scleractinian fishes.

In this study, we identified 26 *BMP* genes in *T. dalaica* based on its whole genome. We also explored the potential roles and regulatory mechanisms of these *BMP* genes in *T. dalaica* through bioinformatics analysis and comparative studies with other vertebrates. These findings lay the foundation for further exploration of *BMP* gene functions in *T. dalaica* and contribute to the understanding of its physiological characteristics at the molecular level, providing support for the protection of germplasm resources in *T. dalaica*.

## Materials and methods

### Materials

The data for the *T. dalaica* transcriptome (Table S1) and genome (PRJNA624716) used in this investigation were derived from the outcomes of our laboratory sequencing. The *T. dalaica* specimens utilized in this study were collected from Dalinuoer Lake (43°22′43″N, 116°39′24″E), Inner Mongolia [16]. For the sequencing of *T. dalaica*, we employed PacBio sequencing and Hi-C technology, which resulted in chromosome-level genomic and transcriptomic data [15]. The de novo assembled genome had a total size of 607.91 Mb, with a contig N50 of 9.27 Mb. The total data volume collected was 126.5 Gb and 106 Gb.

### *BMP* gene identification and sequence analysis

The CDSs and amino acid sequences of four species, *Danio rerio*, *Homo sapiens*, *Cyprinus carpio*, and *Xenopus laevis*, were obtained from publicly available data in the Ensembl database (http://asia.ensembl.org/) and the National Center for Biotechnology Information (NCBI) website (http://www.ncbi.nlm.nih.gov/genbank/). We conducted a BLASTP alignment search against the *T. dalaica* genome database utilizing *BMP* gene protein sequences from these species as query sequences, with an e-value cutoff set at 1e-5. To confirm the accuracy of the identification of the candidate genes in *T. dalaica*, a reverse blast was carried out using the NCBI database. The protein sequences of *T. dalaica*'s *BMP* genes were established by analyzing the intersection of the findings. We assessed the physical and chemical properties of *BMP* family members, including amino acid count, theoretical isoelectric point, molecular weight, and instability index, using TBtools software [17]. Subcellular localization was investigated using the WoLF PSORT website (https://www.genscript.com/wolf-psort.html), while the identification of open reading frames (ORFs) for *BMP* genes was executed via the utilization of ORF Finder (https://www.ncbi.nlm.nih.gov/gorf/gorf.html) [18].

### Multiple sequence comparisons and phylogenetic analysis of *BMP* genes

The amino acid sequences of conserved domains in *BMP* genes belonging to the TGF superfamily were extracted using SAMRT [19]. Multiple sequence alignment was performed using BioEdit [20], aligning the protein sequences of *BMP* genes from *D. rerio, H. sapiens, C. carpio*, and *X. laevis* obtained from the NCBI and Ensembl databases (http://www.ebi.ac.uk/Tools/msa/clustalw2/) [21]. A phylogenetic tree was constructed in MEGA11 using the matched sequences and the neighbor-joining technique [22], with a bootstrap repeat count of 1000. The generated phylogenetic tree was further refined using the ChiPlot online tool (https://www.chiplot.online/) [23]. The *BMP* protein sequences of the four species *D. rerio*, *H. sapiens*, *C. carpio*, and *X. laevis* are given in Table S3.

### Gene nomenclature

The *BMP* genes in *T. dalaica* were identified through a combination of multiple sequence alignment and phylogenetic analyses. *BMP* genes in *T. dalaica* were named based on *BMP* gene sequences from *D. rerio*, *H. sapiens*, *C. carpio*, and *X. laevis*. In instances where multiple *T. dalaica* genes were grouped with the other four species, a Latin numeral suffix was appended to each gene name. The final nomenclature of the *BMP* genes in *T. dalaica*, along with that of the selected species, can be found in Table 1.

**Table 1:** *BMP* gene families in the genomes of the five vertebrates

*Triplophysa dalaica*	*Danio rerio*	*Xenopus laevis*	*Cyprinus carpio*	*Homo sapiens*
26	22	16	44	17
*BMP1a*	*BMP1a*	*BMP1*	*BMP1a-1/2*	*BMP1*
*BMP1b-1*	*BMP1b*		*BMP1b-1/2*	
*BMP1b-2*				
*BMP2a*	*BMP2a*	*BMP2*	*BMP2a-1/2*	*BMP2*
*BMP2b*	*BMP2b*		*BMP2b-1/2*	
*BMP3a*	*BMP3a*	*BMP3a*	*BMP3a-1/2*	*BMP3a*
*BMP3b-1*	*BMP3b*	*BMP3b*	*BMP3b-1/2*	*BMP3b*
*BMP3b-2*				
*BMP4*	*BMP4*	*BMP4*	*BMP4-1/2*	*BMP4*
*BMP5*	*BMP5*	*BMP5*	*BMP5-1/2*	*BMP5*
*BMP6a*	*BMP6*	*BMP6*	*BMP6-1/2*	*BMP6*
*BMP7a*	*BMP7a*	*BMP7a*	*BMP7a-1/2*	*BMP7*
*BMP7b-1*	*BMP7b*	*BMP7b*	*BMP7b-1/2*	
*BMP7b-2*				
	*BMP8a*	*BMP8a*	*BMP8a-1/2*	*BMP8a*
*BMP9*	*BMP9*	*BMP9*	*BMP9-1/2*	*BMP9*
*BMP10a*	*BMP10a*	*BMP10*	*BMP10a-1/2*	*BMP10*
*BMP10b*	*BMP10b*		*BMP10b-1/2*	
*BMP11-1*	*BMP11*	*BMP11*	*BMP11-1/2*	*BMP11*
*BMP11-2*				
*BMP12*	*BMP12*		*BMP12-1/2*	*BMP12*
*BMP13a*	*BMP13a*	*BMP13*	*BMP13a-1/2*	*BMP13*
*BMP13b*	*BMP13b*		*BMP13b-1/2*	
*BMP14*	*BMP14*	*BMP14*	*BMP14-1/2*	*BMP14*
*BMP15-1*	*BMP15*	*BMP15*	*BMP15-1/2*	*BMP15*
*BMP15-2*				
*BMP16*	*BMP16*		*BMP16-1/2*	

### Synteny analysis

To explore gene duplication events within *T. dalaica*, we conducted an intraspecific collinearity analysis. Additionally, a comparative collinearity analysis map was constructed between *T. dalaica* and two additional osteichthyan species, *D. rerio,* and *C. carpio*. Genomic data and annotation information for *D. rerio* and *C. carpio* were obtained from the NCBI and Ensembl databases, respectively. For both intraspecific and interspecific collinearity analyses, we used TBtools software for analysis and diagramming [17].

### Chromosomal localization and gene structure analysis of the *BMP* genes

The chromosomal localization of *T. dalaica BMP* genes was ascertained by importing the complete genome annotation file of *T. dalaica* into TBtools software in conjunction with the *BMP* gene sequence data [17]. Subsequently, the physical positions of the chromosomes corresponding to the *BMP* genes were visualized.

Predictions of the secondary structure elements of *BMP* protein sequences, including α-helices, β-turns, extended strands, and disordered coils, were made using SOPMA (http://pbil.ibcp.fr/) [24]. Subcellular localization prediction of *BMP* protein sequences was performed using Cell-PLoc2.0 http://www.csbio.sjtu.edu.cn/bioinf/Cell-PLoc-2/ [25]. Information on *BMP* gene exons and introns was extracted from the *T. dalaica* whole-genome annotation file, and visualization was carried out using TBtools (http://tbtools.bioinfodata.com/) [17].

### Analysis of conserved motifs and conserved structural domains of the *BMP* genes

MEME (http://meme-suite.org/) was used to predict protein motif patterns in the *T. dalaica BMP* gene family. The maximum motif value was set to 10, with a width range of 6 to 50. The results were visualized using TBtools. Conserved domain structures of *BMP* genes were predicted using the NCBI-Batch-CD-search tool (https://www.ncbi.nlm.nih.gov/Structure/bwrpsb/bwrpsb.cgi) and the SMART tool (http://smart.embl-heidelberg.de). TBtools and the *BMP* protein phylogenetic tree were used for visualization of conserved *BMP* protein motifs and domains [17].

### GO enrichment analysis of *BMP* genes

GO enrichment analysis of the *BMP* genes was conducted using assembled transcripts obtained from whole-genome sequencing of *T. dalaica*. The basic unit of GO enrichment was term, and all differently expressed genes were mapped to each term in the Gene Ontology database (http://www.geneontology.org/) to obtain the number of genes in each term. Apply chi-square test or hypergeometric test to identify GO terms that are significantly enriched in differentially expressed genes compared to the entire genome background. Determine the main biological functions performed by the differentially expressed genes through significant GO functional enrichment analysis.

### Expression patterns of *BMP* genes

The original image data derived from sequencing were converted into sequence data via base calling, yielding raw reads, which were saved in fastq file format. We used Trimmomatic software (http://www.usadellab.org/cms/index.php?page=trimmomatic) to filter the raw data, remove reads with adaptors, discard reads with more than 10% 'N' bases, and eliminate reads where more than 50% of bases had a quality score less than 20 [26]. Afterward, we applied FastQC software (http://www.bioinformatics.babraham.ac.uk/projects/fastqc) for quality control of the clean data, and ultimately, Trinity software was used for the assembly of these clean data. The transcriptome assembled by Trinity was utilized as the reference sequence (ref), onto which clean reads of each sample were mapped. We implemented the RSEM software for this process, with the Bowtie2 parameter set to a mismatch of 0 (the default Bowtie2 parameter) [27]. RSEM provided statistical analysis of the bowtie alignment results, furnishing the read count numbers for each gene in every sample [28]. These counts were subsequently subjected to TPM (transcripts per million) conversion—a normalized value indicating the quantity of transcripts for a specific gene or transcript per million total transcripts within the sample—which facilitated the analysis of gene expression levels. Ultimately, this analysis resulted in the quantified abundances of *BMP* gene transcripts. A heatmap of gene expression was generated using the online tool ChiPlot (https://www.chiplot.online/) [23, 29].

**Fig. 1:**
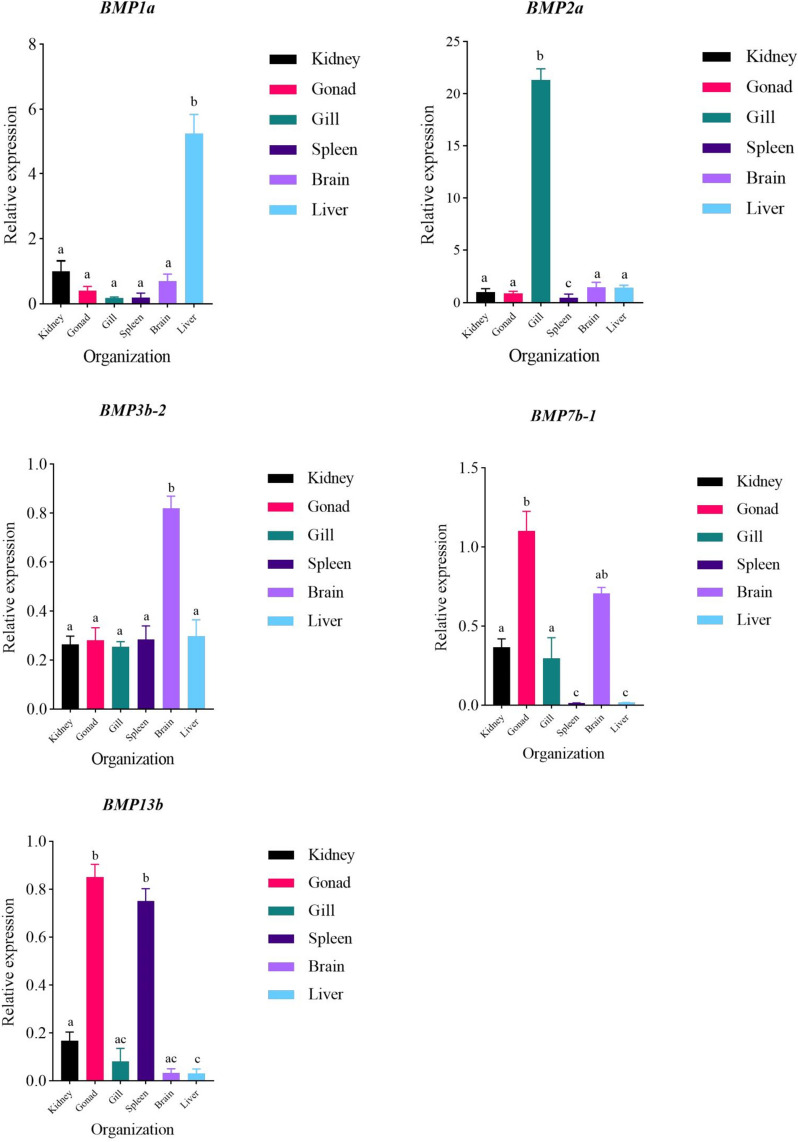
Expression of different genes in each tissue. **a**,**b**,**c** Three letters and the same letter indicate no significant difference, and different letters indicate significant differences

We also performed real-time fluorescence quantitative PCR (qRT‒PCR) analysis of selected tissues and genes to verify the accurate transcriptome abundance analysis of the gene expression trends. Three specimens of *T. dalaica* from the saline-alkali waters of Dali Lake (Inner Mongolia) were collected, and RNA was extracted from six tissues—brain, liver, spleen, gonad, kidney, and gill—followed by cDNA synthesis and RT‒PCR. Specific primers for certain members of the *BMP* gene family in *T. dalaica* were designed using Primer 5.0 (Table S2). The qRT‒PCR experiments were conducted using the TransStart Top Green qPCR SuperMix kit. The amplification system consisted of a 10 μL reaction mixture with 1 μL cDNA, 0.2 μL forward primer, 0.2 μL reverse primer, 5 μL TransStart Top Green qPCR SuperMix, and 3.6 μL ddH2O. The experiments were performed on a LightCycler 96 Real-Time PCR System with three biological and three technical replicates. β-actin was used as the internal control gene, and the data were processed using the 2^−△△Ct^ method, where △△Ct = (average Ct value of the target gene in treated samples—average Ct value of the internal control gene in treated samples)—(average Ct value of the target gene in control samples—average Ct value of the internal control gene in control samples) [30]. With the kidney group set as 1 for the same gene, the relative expression levels were calculated and graphed in GraphPad Prism 9. The relative expression levels of all the genes were analyzed via one-way ANOVA followed by Duncan's multiple range test, with P < 0.05 indicating statistical significance. The final results are presented in Fig. 1.

## Results

### *BMP* gene identification and characterization

Utilizing the whole-genome data of *T. dalaica*, we conducted a comparison to eliminate duplicate sequences, considering other species such as *D. rerio*, *H. sapiens*, *C. carpio*, and *X. laevis*. Furthermore, by integrating information from relevant literature [31, 32], we successfully identified a total of 26 *BMP* gene sequences (*BMP1a*-*BMP16*) in *T. dalaica*, which were subsequently categorized into five groups: *BMP1/3/11/15* (Group I), *BMP12/13/14* (Group II), *BMP2/4/16* (Group III), *BMP9/10* (Group IV), *and BMP5/6/7* (Group V).

These 26 genes were discovered within the *T. dalaica* genome and are described in Table 1. We also analyzed the physicochemical characteristics of the 26 protein sequences, which are presented in Table 2. The study revealed that the open reading frame (ORF) lengths of the 26 *BMP* genes varied from 738 (*BMP15-1*) to 3069 (*BMP1b-1*) base pairs (bp), with the number of exons ranging from 1 to 21. The predicted isoelectric points ranged from 4.97 to 9.94, with 11 sequences exhibiting acidity and 15 showing alkalinity. Interestingly, most *BMP* proteins in *T. dalaica* tend to precipitate under highly alkaline conditions, possibly due to the alkaline environment of the species. The predicted code sequence of the *BMP* gene family varies in the number of amino acids, ranging from 245 (*BMP15-1*) to 1022, with relative molecular masses varying from 28.07 kDa to 115.35 kDa. Notably, the average affinity coefficients for *T. dalaica BMP* gene family proteins were consistently negative, indicating affinities for each of these proteins. Additionally, a majority of the *BMP* proteins displayed a genetic instability index above 40, indicating stability, whereas those considered unstable had an index less than 30.

**Table 2:** Characteristics of *BMP* Gene Family Members in the Genome of *Triplophysa dalaica*

Name	ORF (bp)	Exon numbers	Isoelectric Point	Molecular weight (kDa)	Amino Acid	Instability Index	Aliphatic index	Average coefficient of hydrophilicity	Subcellular localization	NCBI accession number
*BMP1a*	2931	20	6.22	109.77	976	40.68	63.44	-0.547	plas	OR733219
*BMP1b-1*	3069	21	5.62	115.35	1022	42.42	62.67	-0.589	extr	OR733217
*BMP1b-2*	2928	20	5.57	108.73	975	44.43	67.6	-0.426	plas	OR733218
*BMP2a*	1176	2	8.58	43.93	391	57.04	80.43	-0.427	plas	OR733221
*BMP2b*	1248	2	8.61	46.97	415	53.64	78.94	-0.524	plas	OR733220
*BMP3a*	1356	3	9.36	51.76	451	53.45	77.38	-0.566	extr	OR733224
*BMP3b-1*	1341	3	9.08	51.27	446	51.25	80	-0.462	extr	OR733222
*BMP3b-2*	1314	3	9.07	49.72	437	54.51	79.43	-0.397	plas	OR733223
*BMP4*	1206	2	6.68	45.8	401	49.64	84.56	-0.528	plas	OR733225
*BMP5*	1338	7	9.22	50.41	445	46.86	74.76	-0.461	mito	OR733226
*BMP6a*	1254	7	8.07	48.09	417	53.05	77.19	-0.481	extr	OR733227
*BMP7a*	1278	7	7.25	48.49	425	49.41	79.13	-0.376	extr	OR733230
*BMP7b-1*	1284	7	6.19	48.7	427	48.51	84.05	-0.292	plas	OR733228
*BMP7b-2*	1284	7	6.19	48.7	427	48.51	84.05	-0.292	plas	OR733229
*BMP9*	1197	2	5.57	45.77	398	38.65	84.42	-0.44	cyto	OR733231
*BMP10a*	1317	3	6.26	50.15	438	47.32	75.64	-0.58	mito	OR733233
*BMP10b*	1422	2	4.97	52.66	473	43.7	79.34	-0.454	extr	OR733232
*BMP11-1*	1113	3	5.64	41.44	370	62.39	79.32	-0.419	extr	OR733234
*BMP11-2*	1128	3	6.22	42.06	375	56.11	80.61	-0.373	extr	OR733235
*BMP12*	1290	2	9.54	48.03	429	56.84	79.28	-0.457	nucl	OR733236
*BMP13a*	1224	2	9.15	46.39	407	53.38	76.93	-0.545	extr	OR733238
*BMP13b*	1248	2	9.42	47.39	415	56.8	76.39	-0.543	extr	OR733237
*BMP14*	1530	2	9.94	57.19	509	38.97	80.47	-0.44	E.R	OR733239
*BMP15-1*	738	1	9.05	28.07	245	53.49	71.14	-0.56	nucl	OR733240
*BMP15-2*	1287	2	8.45	49.87	428	57.05	79.67	-0.57	plas	OR733241
*BMP16*	1275	2	9.43	47.34	424	52.12	79.98	-0.45	extr	OR733242

Abbreviations used in this table: *plas* Plasma membrane, *extr* Extracell, *mito* Mitochondria, *cyto* Cytoplasm, *nucl* Nucleus, *E.R.* Endoplasmic reticulum

Subcellular localization findings, as presented in Table 2, revealed that 11 *BMP* proteins were found in the extracellular plasma, while 9 *BMP* proteins were located in the plasma membrane. The remaining *BMP* proteins were detected in various locations, including the cell nucleus, fibromyalgia, endothelial plasma, and cellulose.

### *BMP* Genetic System Development Analysis

**Fig. 2:**
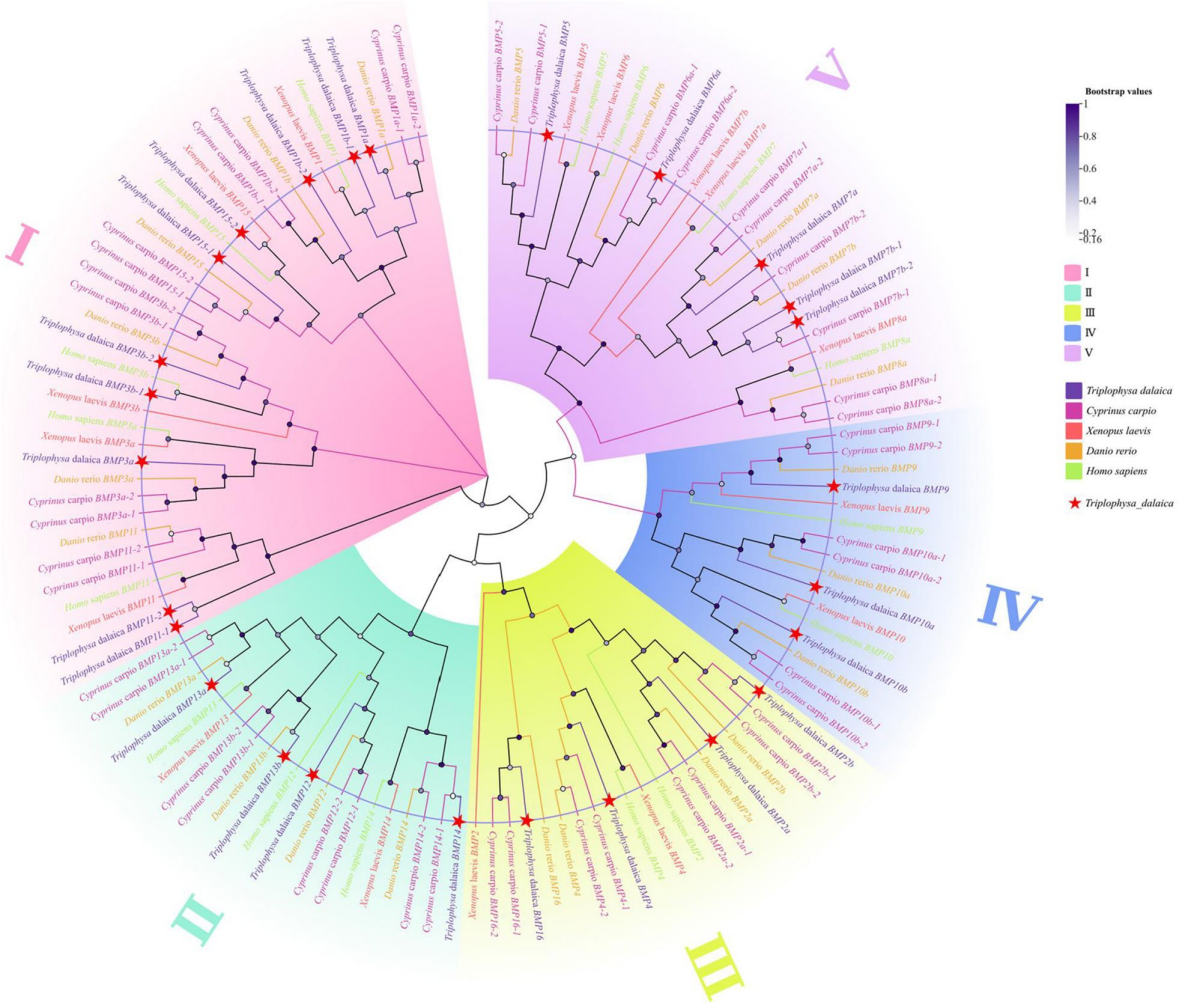
Phylogenetic analysis of the *BMP* genes of *T. dalaica* and selected species. The different colors on the outer ring in the figure represent the different groups, the different species are represented by different colors, and the *BMP* gene of *T. dalaica* is marked by a red star

To explore the evolutionary correlation of the *BMP* gene family in *T. dalaica*, we generated a comprehensive phylogenetic tree using protein sequences derived from *D. rerio*, *H. sapiens*, *C. carpio*, and *X. laevis* (Fig. 2). The phylogenetic tree analysis revealed distinct clustering of the *BMP* gene family in *T. dalaica* and other species into five groups: *BMP1/3/11/15* (Group I), *BMP12/13/14* (Group II)*, BMP2/4/16* (Group III), *BMP9/10* (Group IV), *and BMP5/6/7* (Group V). Notably, the *BMP8a* protein sequences of *D. rerio*, *H. sapiens*, *C. carpio*, and *X. laevis* formed a distinct branch. The majority of the *BMP* genes in *T. dalaica* were categorized into Group I. Phylogenetic analysis revealed a notable level of similarity between *T. dalaica* and two other species, *D. rerio* and *C. carpio*, both of which belong to the Cypriniformes order, in contrast to humans and the amphibian *X. laevis* (Fig. 2).

### *BMP* gene collinearity analysis

**Fig. 3:**
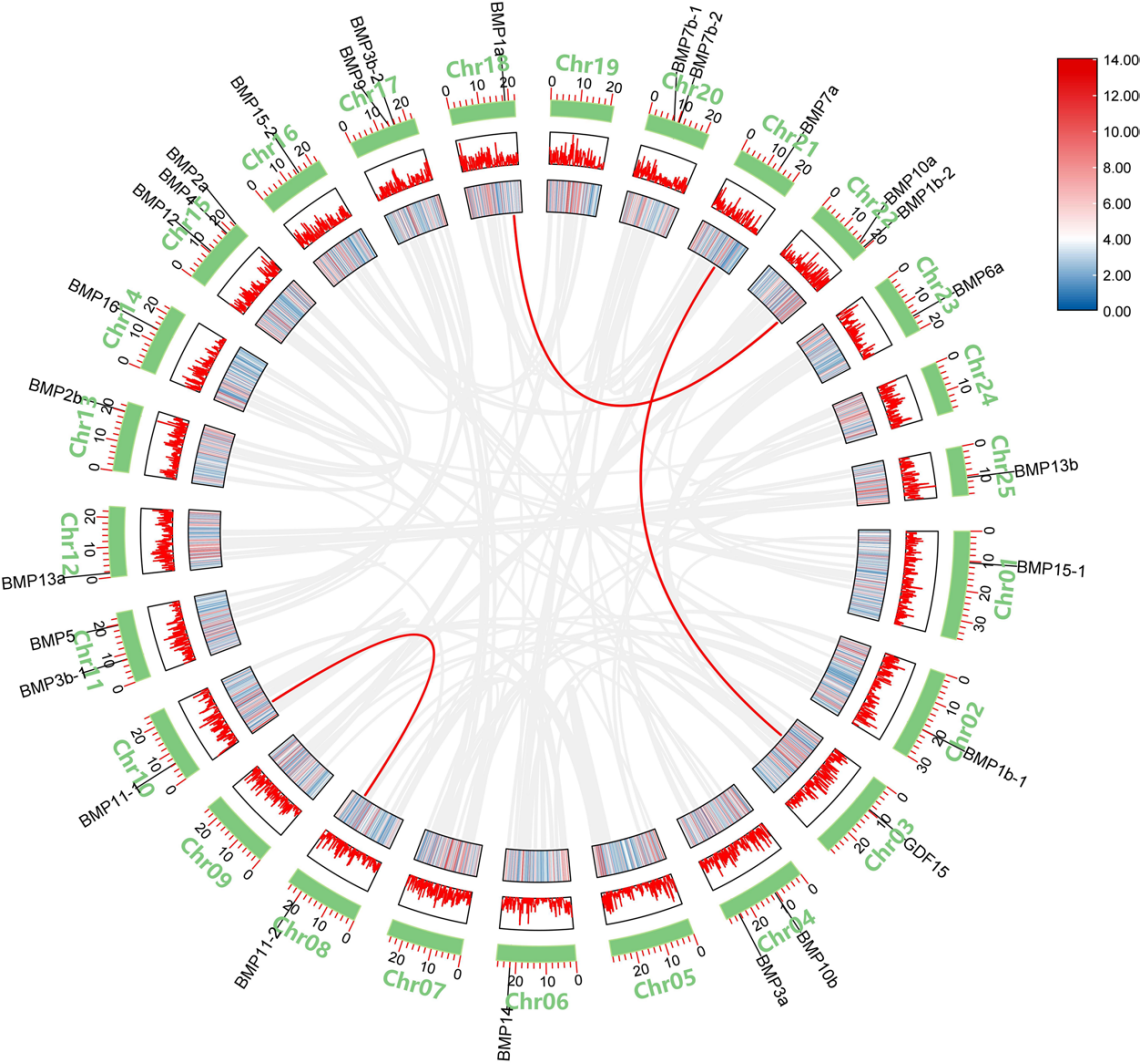
Analysis of *BMP* gene covariance in *T. dalaica.* The gray line indicates the genome covariance of *T. dalaica*, and the colored line connecting the *BMP* genes indicates the duplication of *BMP* genes in *T. dalaica*. The position of the *BMP* genes on the chromosomes is indicated by the short black line, and the density of the genes on each chromosome is shown at the same time

**Fig. 4:**
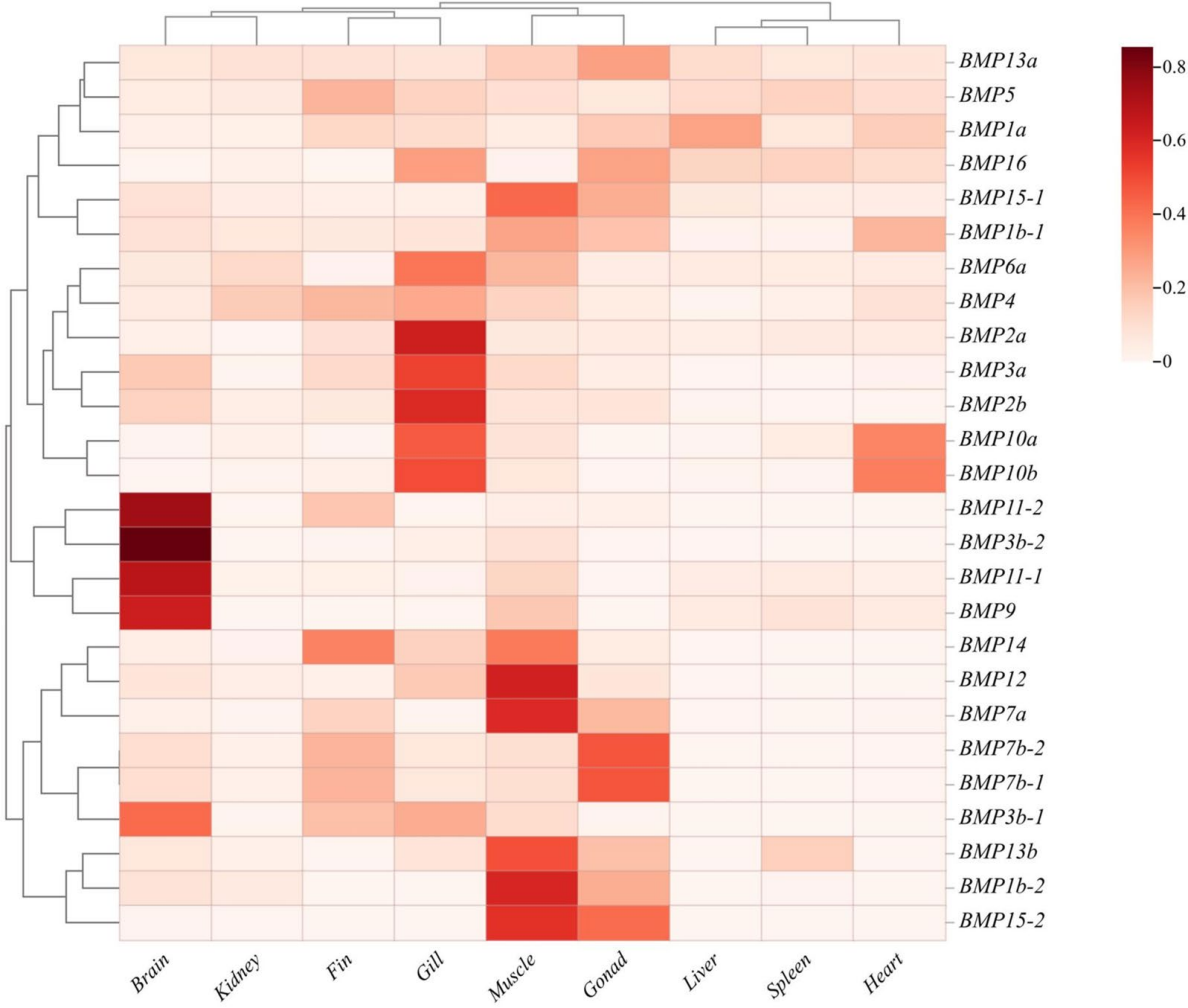
Analysis of the *BMP* gene expression pattern in *T. dalaica*. The square color scale of the heatmap indicates the TPM values after row normalization; the specific values are marked in the color block, and the maximum value is indicated in red

**Fig. 5:**
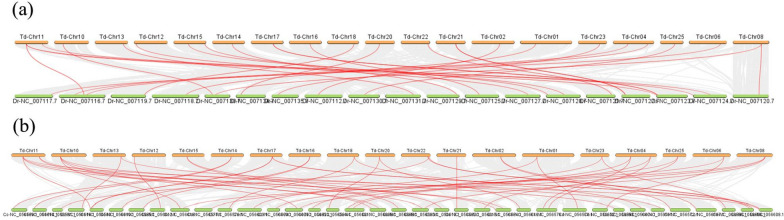
Interspecies covariance analysis of *T. dalaica* with *D. rerio* and *C. carpio.* **a** represents the covariance results of *T. dalaica* with *D. rerio*, **b** represents the covariance results of *T. dalaica* with *C. carpio*, gray lines represent the covariance blocks of *T. dalaica* with *D. rerio* and *C. carpio*, and red lines represent the highlighted *BMP* gene blocks. The abbreviations "Td", "Dr" and "Cc" denote *T. dalaica*, *D. rerio* and *C. carpio*, respectively

We identified three occurrences of gene segment duplication in *T. dalaica*, all resulting from gene fragment duplications; these duplications were located on chromosomes 3 and 21, 8 and 10, and 18 and 22 (Fig. 3). *BMP1a* and *BMP1b-2* underwent gene segment duplications and belonged to the same gene group, with their encoded proteins exhibiting activities similar to those of *BMP11-1* and *BMP11-2*. Interestingly, segmental duplication of *BMP7a* occurred alongside the non-*BMP* gene. The *BMP7* gene belongs to the TGF-β superfamily, and Fig. 4 shows that *BMP7a* is abundantly expressed in fins, muscles and gonads, which suggests that *BMP7a* may be involved in the realization of locomotor function in *T. dalaica* and influence sex differentiation in *T. dalaica*. Figure 3 shows that *BMP7a* is duplicated with the GDF15 gene, possibly because it belongs to the same TGF-β superfamily as the *BMP7a* gene is, and the GDF15 gene has an assisting role in the physiological function of *BMP7a*. Since *T. dalaica*, *D. rerio*, and *C. carpio* are all Cypriniformes, we explored interspecies collinearity among the three species and revealed a significant collinearity link. Most of the *T. dalaica BMP* genes exhibited collinearity with *D. rerio* and *C. carpio* genes (Fig. 5).

### Chromosomal localization and gene structure analysis of the *BMP* genes

**Fig. 6:**
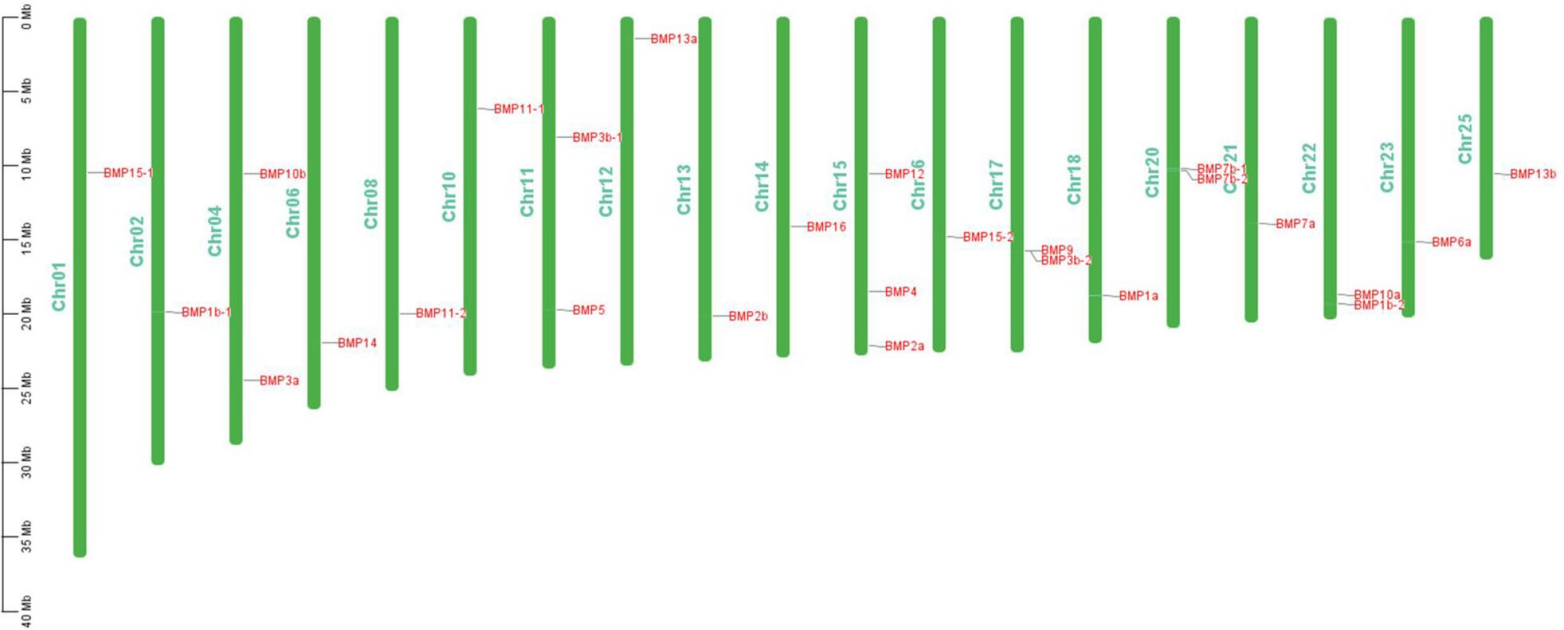
Chromosomal locations of *BMP* genes in the *T. dalaica* genome

We determined the chromosomal location of the *BMP* gene family in *T. dalaica* using TBtools software [17], and the results are presented in Fig. 6. This investigation revealed that the 26 *BMP* genes in *T. dalaica* were dispersed among 19 chromosomes (1, 2, 4, 6, 8, 10, 11, 12, 13, 14, 15, 16, 17, 18, 20, 21, 22, 23, 25). Intriguingly, an overrepresentation of *BMP* genes was observed on chromosomes 4, 11, and 15, suggesting a non-uniform and stochastic distribution pattern within the species. Additionally, we observed close proximity of *BMP9* and *BMP3b-2* on chromosome 17, suggesting the potential formation of a gene cluster resulting from early gene duplication events (Fig. 6) [33, 34].

**Fig. 7:**
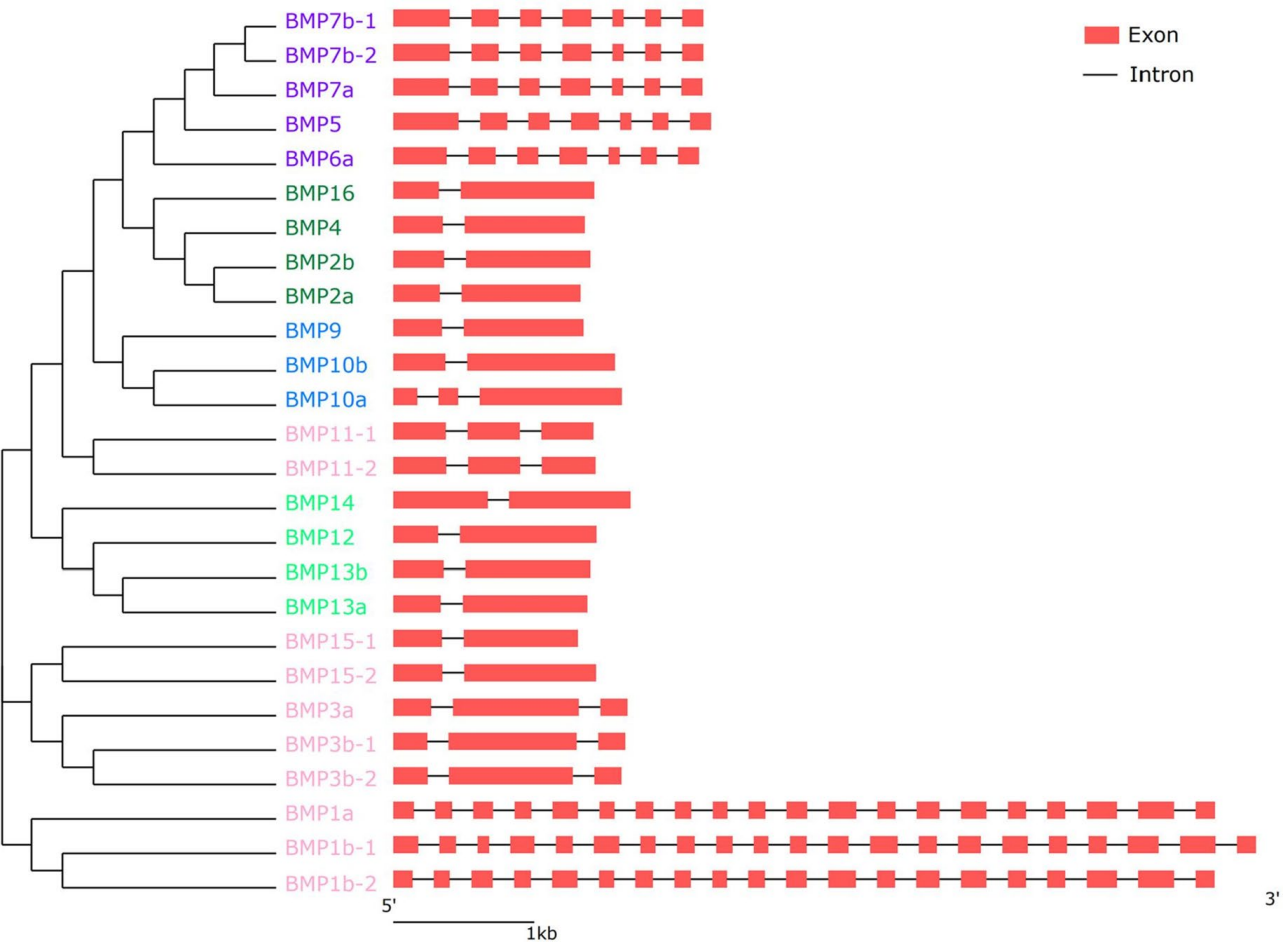
Gene structure of the *BMP* gene of *T. dalaica*. The phylogenetic tree on the left was constructed in MEGA11 based on the neighbor‒joining method, with a bootstrap value of 1000, and different colors represent different groups

To investigate the structural variation of the *BMP* gene family, we used TBtools to visualize the exon‒intron architectures of the 26 *BMP* genes (Fig. 7) [17]. The results showed that *BMP1b-1* had the highest number of exons (21), while the number of other exons ranged from 2 to 21. We also generated an NJ phylogenetic tree to examine *T. dalaica BMP* gene protein sequences and assess gene arrangement. *BMP* genes with similar structural properties were grouped in the evolutionary tree. These findings shed light on the diversity and evolution of the *BMP* gene family. Furthermore, the secondary structure prediction indicated that *T. dalaica BMP* gene proteins contain α-helices, β-turns, extended strands, and irregular coils (Table 3). The percentage of α-helices ranged from 11.17% (*BMP1a*) to 32.41% (*BMP9*), β-turns from 1.23% (*BMP13a*) to 5.84% (*BMP1a*), extended strands from 13.05% (*BMP12*) to 27.77% (*BMP1a*), and irregular coils from 46.73% (*BMP9*) to 62.45% (*BMP15*–*1*). The secondary structure prediction suggested that irregular coils are the predominant structural component of *T. dalaica BMP* genes.

**Table 3:** Secondary structure of the *BMP* gene protein from *Triplophysa dalaica*

Name	Alpha helix	Beta turn	Extended strand	Random coil
*BMP1a*	11.17%	5.84%	27.77%	55.23%
*BMP1b-1*	12.43%	5.38%	27.40%	54.79%
*BMP1b-2*	13.03%	5.33%	27.59%	54.05%
*BMP2a*	24.04%	1.28%	19.18%	55.50%
*BMP2b*	22.65%	3.13%	18.80%	55.42%
*BMP3a*	26.61%	5.32%	15.74%	52.33%
*BMP3b-1*	25.34%	2.69%	16.37%	55.61%
*BMP3b-2*	29.98%	4.58%	15.33%	50.11%
*BMP4*	20.95%	1.75%	21.45%	55.86%
*BMP5*	23.37%	2.47%	18.88%	55.28%
*BMP6a*	26.62%	3.12%	17.27%	53.00%
*BMP7a*	24.47%	4.71%	18.82%	52.00%
*BMP7b-1*	22.72%	4.22%	21.55%	51.52%
*BMP7b-2*	22.72%	4.22%	21.55%	51.52%
*BMP9*	32.41%	2.76%	18.09%	46.73%
*BMP10a*	24.89%	2.51%	18.26%	54.34%
*BMP10b*	30.66%	3.59%	14.16%	51.59%
*BMP11-1*	23.51%	2.70%	21.35%	52.43%
*BMP11-2*	21.07%	2.93%	22.67%	53.33%
*BMP12*	25.64%	3.73%	13.05%	57.58%
*BMP13a*	24.57%	1.23%	14.00%	60.20%
*BMP13b*	25.30%	2.41%	13.73%	58.55%
*BMP14*	23.77%	2.55%	14.93%	58.74%
*BMP15-1*	16.33%	2.04%	19.18%	62.45%
*BMP15-2*	27.80%	1.87%	13.32%	57.01%
*BMP16*	20.99%	2.59%	17.45%	58.96%

### Analysis of conserved motifs and structural domains of the* BMP* genes

**Fig. 8:**
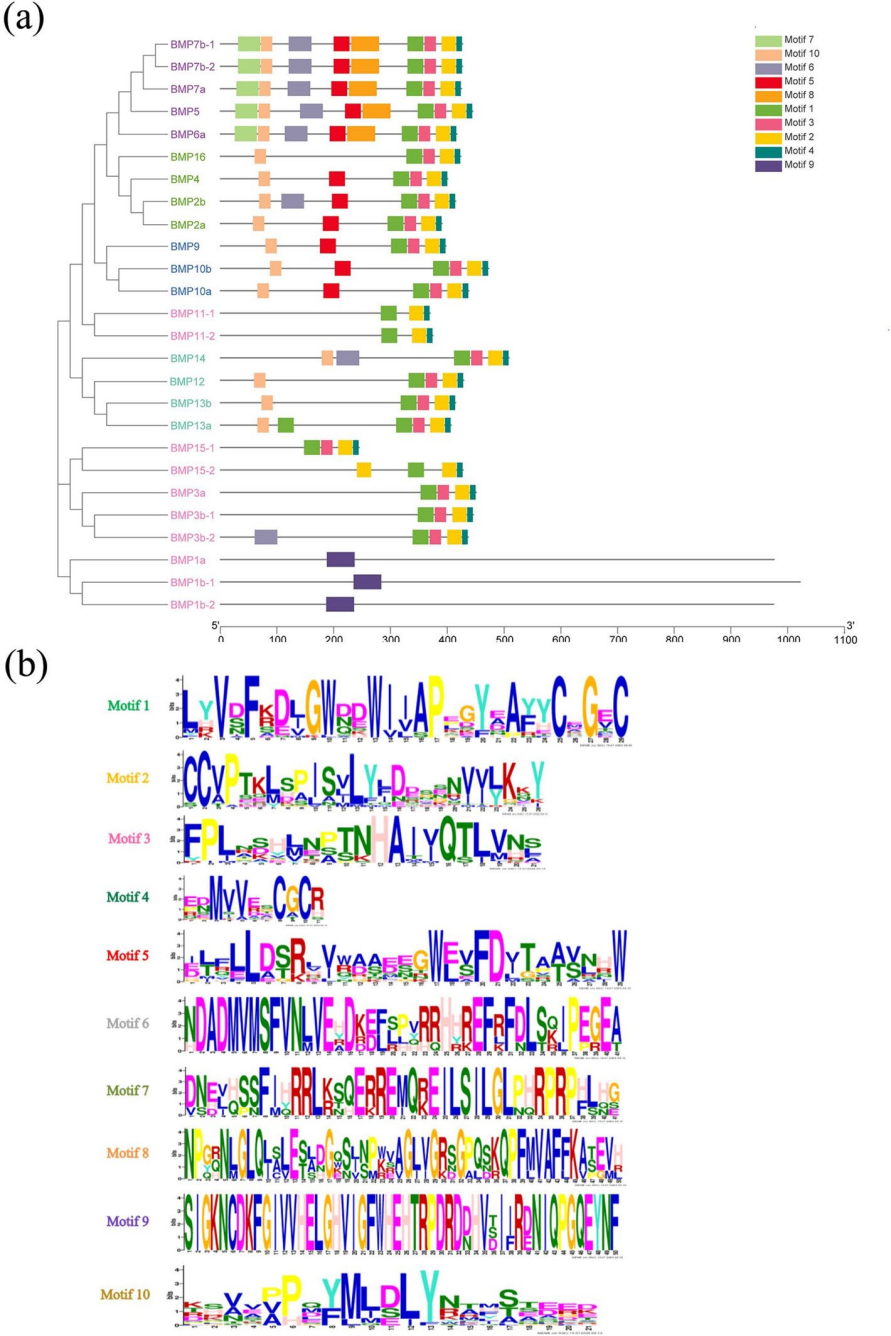
Conserved motifs of the *BMP* gene of *T. dalaica*. **a** The phylogenetic tree on the left was constructed in MEGA11 based on the neighbor‒joining method, with a bootstrap value of 1000, and different colors represent different groups. **b** All motifs were identified by the MEME database, and different colored blocks represent different motifs

We utilized the MEME online program to investigate the conserved protein motifs of the *BMP* gene family in *T. dalaica*, resulting in the identification of ten conserved protein motifs (Fig. 8(b)). Notably, motif 1 featured a conserved sequence of LYVDFKDJGWDDWIIAPEGYEAYYCEGEC, motif 2 was characterized by a conserved sequence of CCVPTKLSPISVLYLDDSENVVLKKY, and motif 4 exhibited a conserved sequence of EBMVVESCGCR, all with a length of 11 amino acids. The analysis revealed that genes with closer evolutionary relationships displayed more similar motif architectures. While motif 9 was found to be exclusively conserved in *BMP1a*, *BMP1b-1*, and *BMP1b-2*, motifs 1, 2, and 4 were detected in all the other family members, underscoring their notable conservation within the core functional domains of the *BMP* genes (Fig. 8). The distribution of motifs aligns with the phylogenetic tree, demonstrating that genes within the same subgroup exhibited similar motif patterns (Fig. 8(a)).

**Fig. 9:**
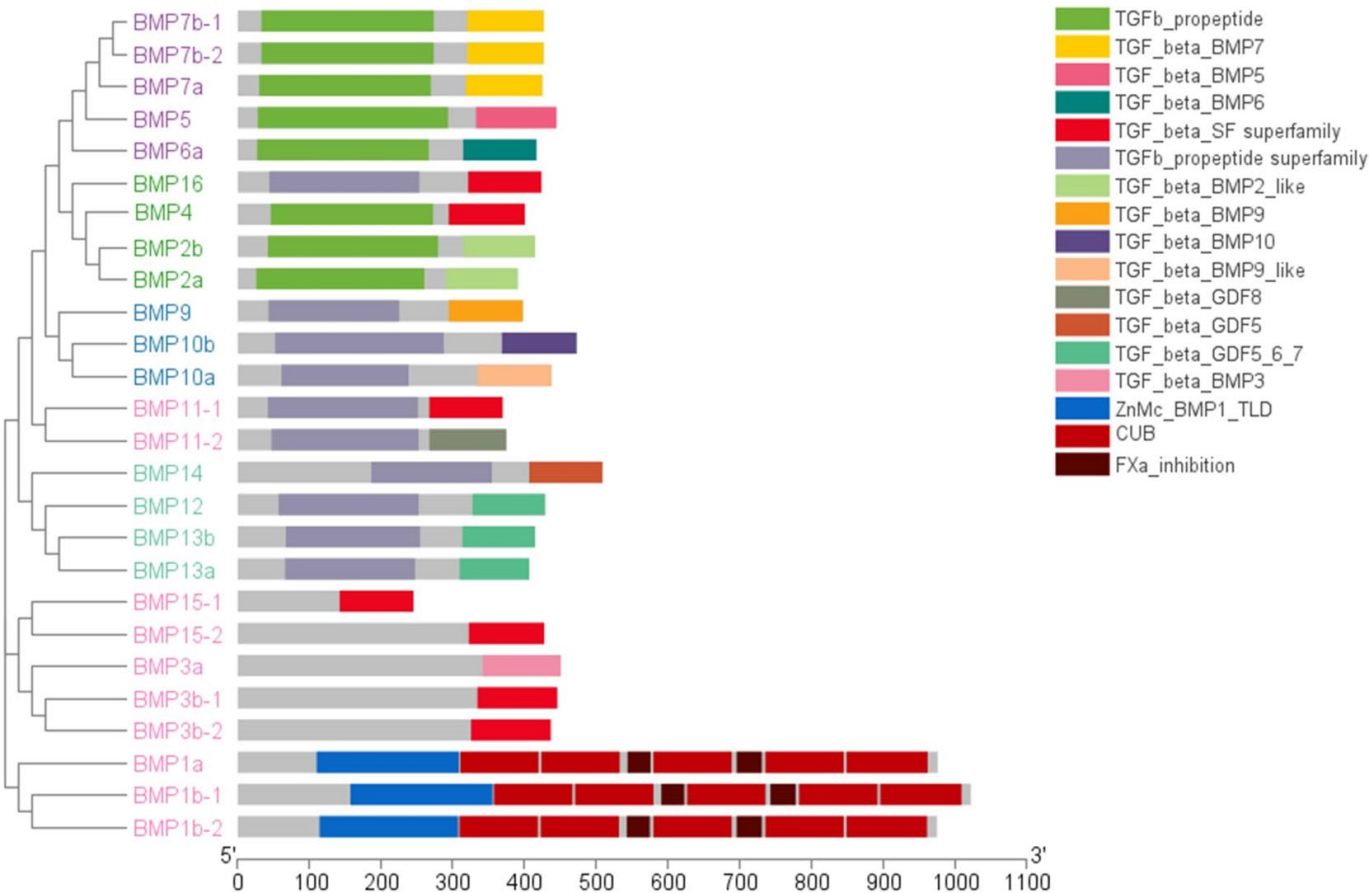
Structural domains of the *BMP* gene of *T. dalaica*. The phylogenetic tree on the left was constructed in MEGA11 based on the neighbor‒joining method, with a bootstrap value of 1000, and different colors represent different groups

Moreover, as depicted in Fig. 9, the *BMP* gene family members in *T. dalaica* that clustered together under the same branch exhibited notable similarities in terms of conserved protein domains. These observations are consistent with the observed distribution patterns and the results of the phylogenetic tree analysis. Notably, the *BMP1a*, *BMP1b-1*, and *BMP1b-2* genes produced proteins with distinct conserved domains ("ZnMc," "CUB," "Fxa"), arising from their unique gene structures. *BMP1*, classified as a zinc-dependent metalloproteinase, does not belong to the TGF-β superfamily [35, 36].

### GO enrichment analysis of the *BMP* genes

We conducted a GO enrichment analysis, and the results presented in Table 4 revealed the involvement of the *BMP* gene family in *T. dalaica* in a total of 15 distinct GO pathways. Among these pathways, eight were associated with molecular functions (F), six were related to biological processes (P), and one was linked to cellular components (C). Notably, the GO pathways related to molecular functions were primarily associated with growth and development processes, including activities such as metallopeptidase activity, calcium ion binding, metalloendopeptidase activity, zinc ion binding, growth factor activity, receptor binding activity, and protein domain-specific binding. The GO pathways linked to biological processes predominantly included protein hydrolysis, cellular cytoskeleton organization, cell migration, and cell adhesion. The examination of the *T. dalaica BMP* gene phylogenetic tree (Fig. 7) suggested that the *BMP1/3/11/15* (Group I) subfamily is involved primarily in these biological processes. Most *BMP* genes were enriched in the GO:0008083 pathway, associated with growth factor activity, aligning with the characteristic function of the *BMP* gene family, suggesting that the *BMP1/3/11/15* (Group I) subfamily predominantly plays a role in *T. dalaica*'s growth and development processes.

**Table 4:** GO enrichment analysis of the *BMP* gene family

GO ID	GO Term	Biological Function	genes
GO:0004222	metalloendopeptidase activity	F	*BMP1b-1,BMP1b-2,BMP1a*
GO:0005509	calcium ion binding	F	*BMP1b-1,BMP1b-2,BMP1a*
GO:0006508	proteolysis	P	*BMP1b-1,BMP1b-2,BMP1a*
GO:0008237	metallopeptidase activity	F	*BMP1b-1,BMP1b-2,BMP1a*
GO:0008270	zinc ion binding	F	*BMP1b-2,BMP1a,*
GO:0008083	growth factor activity	F	*BMP2b,BMP2a,BMP3b-1,BMP3b-2,BMP3a,BMP4,BMP5,BMP6a,BMP7b-1,BMP7b-2,BMP7a,BMP9,BMP10b,BMP10a,BMP11-1,BMP11-2,BMP12,BMP13b,BMP13a,BMP14,BMP15-2,BMP16*
GO:0005102	signaling receptor binding	F	*BMP11-1,BMP11-2*
GO:0004064	arylesterase activity	F	*BMP13a*
GO:0007010	cytoskeleton organization	P	*BMP13a*
GO:0016477	cell migration	P	*BMP13a*
GO:0019904	protein domain specific binding	F	*BMP13a*
GO:0098609	cell‒cell adhesion	P	*BMP13a*
GO:0005615	extracellular space	C	*BMP15-1*
GO:0001541	ovarian follicle development	P	*BMP15-1*
GO:0060016	granulosa cell development	P	*BMP15-1*

### Expression analysis

Comprehending gene expression patterns plays a vital role in revealing gene functionality. In our study, we conducted an analysis of *BMP* gene expression data from various tissues of *T. dalaica*, thus shedding light on the expression patterns of members of the *BMP* gene family. The expression of several *BMP* genes in six tissues, *T. dalaica*, brain, liver, spleen, gonad, kidney and gill, was analyzed via real-time fluorescence quantitative PCR to verify the trend in the abundance of the *BMP* gene transcriptome. As shown in Fig. 1, the expression of *BMP1a* was greater in the liver than in the other tissues. *BMP2a* had the highest expression in the gill, *BMP3b-2* had a much greater expression in the brain than in the other tissues, and *BMP7b-1* had the highest expression in the gonad and the second highest expression in the brain. Similarly, the expression of *BMP13b* was greater in the gonad and spleen than in the other tissues. Figure 1 shows that the change in expression of the *BMP* gene in different tissues was the same as the change in expression in terms of transcriptome abundance (Fig. 4), which also indicated that the change in gene expression in the transcriptome abundance analysis in this study was correct. The heatmap revealed that the majority of the *BMP* genes in *T. dalaica* were expressed in the brain, gonads, gills, muscles, and fins, while exhibiting comparatively lower expression levels in the kidney, liver, spleen, and heart tissues. In particular, the brain presented noteworthy expression levels of *BMP11*-*2*, *BMP3b-1*, *BMP3b-2*, *BMP11*-*1*, and *BMP9*, with *BMP3b-2* demonstrating the highest expression. This discovery indicates that *BMP11s* are likely secreted by neurons, suggesting their preference for expression in the brain [33]. In muscle tissues, *BMP* genes are universally expressed, with *BMP12* and *BMP1b-2* exhibiting the highest expression levels. In the gills, *BMP2a*, *BMP3a*, *BMP2b*, *BMP10a*, and *BMP10b* exhibited increased expression levels (Fig. 4). *BMP1a* is highly expressed in the gonads, liver, and heart; *BMP1b-1,* in muscles and gonads; and *BMP1b-2,* in muscles and gonads, suggesting that *BMP*1 is broadly involved in the development of gonads and metabolism in *T. dalaica* (Fig. 4). *BMP3a*, *BMP3b-1*, and *BMP3b-2* are highly expressed in the brain, fins, gills, and muscles, suggesting *the* potential impact of *BMP3* on skeletal plasticity. *BMP11* is highly expressed in the brain, fins, and muscles, suggesting its potential role in the development, differentiation, and tissue formation of the nervous system in *T. dalaica*. *BMP15-1* and *BMP15-2* are highly expressed in muscles and gonads, suggesting that they play a role in reproductive system development. GO enrichment analysis revealed that the *BMP1/3/11/15* (Group I) genes primarily contributed to the growth and development of *T. dalaica*.

*BMP7a*, *BMP7b-1*, and *BMP7b-2* are expressed primarily in the fins, muscles, and gonads. *BMP7a* is almost not expressed in the spleen, and the expression of *BMP7b-1* and *BMP7b-2* in the spleen and liver of *T. dalaica* is also negligible. *BMP12* expression is almost nonexistent in the spleen and heart, suggesting that it plays a minor role in the physiological processes of the spleen and heart in *T. dalaica*. Similarly, *BMP13b* is hardly expressed in the liver, fins, or heart. *BMP14* was also almost not expressed in the spleen or heart. It is hypothesized that these genes do not participate in the physiological functions of the corresponding tissues in *T. dalaica* under normal physiological conditions. However, further research may be needed to determine the specific roles of these genes.

## Discussion

The primary functions of *BMP* genes include the control of osteoblast and chondrocyte development and differentiation and the promotion of bone healing, all of which are crucial for bone production [32, 37, 38]. The *BMP* gene family comprises essential signaling molecules involved in regulating cell division, proliferation, function, and tissue morphogenesis [39]. Furthermore, due to their significance as growth factors, they have a wide range of potential applications in various industries, including skeletal disease therapy and regenerative medicine [40, 41].

Leveraging whole-genome sequencing data collected by our research team, we successfully identified a comprehensive set of 26 *BMP* gene family members in the *T. dalaica* genome. Within this study, we conducted an in-depth analysis encompassing the physicochemical characteristics, subcellular localization, phylogenetic associations, intraspecies and interspecies collinearity, chromosomal mapping, and gene structure of these 26 *BMP* gene family members in *T. dalaica*, as well as the identification of conserved motifs and domains. The classification of these *BMP* genes within the *T. dalaica* genome was determined through the evaluation of sequence homology and evolutionary relationships. We identified five different subgroups, namely, the *BMP 1/3/11/15* (Group I), *BMP 12/13/14* (Group II), *BMP 2/4/16* (Group III), *BM 9/10* (Group IV), and *BMP 5/6/7/8* (Group V) subgroups, which differed from the findings of previous studies of cobia [32]. These findings align with the research outcomes documented in previous studies on carp [33]. Compared to the other species studied (*D. rerio* and *X. laevis*), *T. dalaica* has a relatively complete *BMP* gene family, which includes three *BMP1* genes, three *BMP3* genes, three *BMP7* genes, two *BMP11* genes, and two *BMP15* genes. This difference may be the result of the scleractinian WGD event [34]. This gene duplication results in new copies of genes with the potential for differentiated functions or new functions adapted to new environments, likely due to the specific survival environment of the *T. dalaica* species, which allows these genes to be expressed in large numbers [42, 43]. This phenomenon aligns with the ecological adaptations of scleractinian fishes [44, 45]. Notably, *T. Dalaica* may have undergone a gene deletion event during evolution, as the *BMP8* gene is missing in *T. dalaica* compared to other scleractinian fishes (e.g., zebrafish, carp, etc.) and vertebrates [45].

In *T. dalaica*, the *BMP15* gene is highly expressed in muscles and gonads and plays a significant role in the development of the reproductive system [46, 47]. The *BMP11* gene is produced by neurons and is significantly expressed in the brain (Fig. 4) [33]. Most of the *BMP* genes belong to *BMP1/3/11/15* (Group I) and are likely influenced by the specific environmental conditions in which *T. dalaica* thrives [48]. The results of our phylogenetic analysis revealed that *T. dalaica*, *D. rerio*, and *C. carpio*, all of which belong to the Cypriniformes order, share a close evolutionary relationship and form a distinct cluster. This separation distinguishes them from amphibians, such as *X. laevis,* and mammals, such as *H. sapiens*. These findings align with the taxonomic criteria for species classification, indicating that *BMP* genes in *T. dalaica* have maintained a high degree of conservation throughout their evolutionary history. The conserved base sequence, analysis of structurally conserved domains, and evolutionary relationships among *T. dalaica BMP* genes further suggest that *BMP* genes within the same subgroup share comparable base sequences, types of structural domains, and distribution patterns [49, 50]. This observation aligns with the common amino acid sequences shared among members of the TGF-β superfamily. Notably, there was repetition and fragmentation of the genes *BMP1a* and *BMP1b*-2 and of *BMP11-1* and *BMP-11–2*, which belong to the same gene group. Notably, this phenomenon was also observed for genes outside the *BMP* group, including *BMP7a*. Furthermore, a Gene Ontology (GO) enrichment analysis of the *BMP* genes revealed that, except for *BMP1a*, *BMP1b-1*, and *BMP1b-2*, the remaining *BMP* genes were associated with the GO pathway related to growth factor activity. By analyzing the expression patterns of *BMP* genes, we observed that genes within the *BMP1/3/11/15* subgroup (Group I) exhibited relatively high expression levels in most tissues. This finding suggested the significant role of these genes in the growth and development of *T. dalaica*. These findings are consistent with prior studies, such as those conducted in cobia and carp, where *BMP15* exhibited high expression across multiple tissues and is generally regarded as crucial for female fertility [32]. *T. dalaica* lives in high-altitude and saline-alkaline environments, leading to the development of unique morphological and physiological characteristics adapted to extreme conditions. The genes *BMP1*, *BMP13*, *BMP15*, and *BMP16* are highly expressed in the gonads and likely play key roles in reproductive development and regulating sex ratios to adapt to harsh environments. *BMP2*, *BMP3*, *BMP4*, *BMP6*, *BMP10*, and *BMP16* are highly expressed in the gills, which is speculated to be a result of the environmental adaptation of *T. dalaica*, which involves the evolution of more efficient gills to obtain more oxygen.

## Conclusion

In this research, we identified a total of 26 bone morphogenetic protein (*BMP*) genes within the genome of *T. dalaica*. Our phylogenetic and covariance analyses revealed that *T. dalaica* exhibited the closest genetic relationship to *D. rerio* and C. carpio, revealing strong covariance. We further examined the *BMP* genes in *T. dalaica* and identified instances of gene duplication, which can likely be attributed to whole-genome duplication (WGD) events. Upon scrutinizing the gene expression patterns, we noticed that the duplicated *BMP* genes in *T. dalaica* exhibited elevated expression levels. Furthermore, through extensive Gene Ontology (GO) enrichment analysis, it became evident that the *BMP1/3/11/15* genes (Group I) played a primary role in the growth and development of *T. dalaica*. This study contributes to a deeper understanding of *BMP* gene family member expression patterns in high-altitude, high-salinity environments and provides valuable insights for future research on the *BMP* gene family in bony fishes.

### Supplementary Information

**Additional file 1: Table S1**. *Triplophysa dalaica* transcriptome data used in this study.

**Additional file 2: Table S2**. Primers used for qRT‒PCR of the *BMP* gene family.

A**dditional file 3: Table S3**. NCBI accession numbers for the *BMP* protein sequences of the four species.

## Data Availability

The datasets supporting the results of this article are included within manuscript and available on request (Dr. Chuanjiang Zhou). The *Triplophysa dalaica* genome DNA sequencing data have been deposited into the NCBI Sequence Read Archive under BioProject: PRJNA624716 (https://academic. oup.com/gbe/article/13/8/evab153/6311268). The datasets generated and analysed during the current study are available in the NCBI GenBank: OR733217-OR733242 (Data will be released when the manuscript published).

## Abbreviations

GO: Gene Ontology
CDS: Coding sequence
F: Molecular Functions
P: Biological Processes
C: Cellular Components
WGD: Whole Genome Duplication
TPM: Transcripts Per Kilobase of exon model per Million mapped reads
NJ: Neighbor-Joining Algorithm
