# Supplementary material for: Genome-wide identification and structural analysis of the BMP gene family in Triplophysa dalaica
Source: BMC Genomics. 2024 Feb 19;25:194. doi: 10.1186/s12864-024-10049-z (PMC10875767; doi:10.1186/s12864-024-10049-z)
Supplement: Supplementary file 1 — Additional file 1: Table S1. Triplophysa dalaica transcriptome data used in this study. [file 12864_2024_10049_MOESM1_ESM.docx]

**Table S1** *Triplophysa dalaica* transcriptome data used in this study

| Organisation | NCBI accession number | Size | GC Content | Reference |
| --- | --- | --- | --- | --- |
| Gonad | SRX8097854 | 2.4G | 46.20% | [15] |
| Kidney | SRX8097853 | 2.3G | 45.80% | [15] |
| Gill | SRX8097852 | 2.4G | 46.10% | [15] |
| Spleen | SRX8097851 | 2.3G | 44.40% | [15] |
| Brain | SRX8097850 | 2.4G | 44.20% | [15] |
| Liver | SRX8097849 | 2.3G | 45.50% | [15] |
| Fin | SRX8097846 | 2.2G | 46.00% | [15] |
| Heart | SRX8097845 | 2.3G | 45.40% | [15] |
| Muscle | SRX8097844 | 2.2G | 39.30% | [15] |

**Note:** References can be found in the main text citation section [15].
