# Supplementary material for: Genome-wide identification and structural analysis of the BMP gene family in Triplophysa dalaica
Source: BMC Genomics. 2024 Feb 19;25:194. doi: 10.1186/s12864-024-10049-z (PMC10875767; doi:10.1186/s12864-024-10049-z)
Supplement: Supplementary file 2 — Additional file 2: Table S2. Primers used for qRT‒PCR of the BMP gene family. [file 12864_2024_10049_MOESM2_ESM.docx]

**Table S2** Primers used for qRT‒PCR of the *BMP* gene family

| Gene name | Forward primer (5'-3') | Reverse primer (5'-3') |
| --- | --- | --- |
| *β-actain* | AAGCCGGATTTGCTGGAGA | CGATGGGGTATTTCAGGGTCA |
| *BMP2a* | CAGGACTTGTACTCGATGCAC | CGTTGGCTGGAGACGTTAT |
| *BMP3b-2* | GACATTGCCATAAACGAACC | GGACCCTCTAAACCCCTCAT |
| *BMP7b-1* | CACAGCCACCAGTAACCACT | CCTTTGGCGGTTTTAGAGCG |
| *BMP13b* | CATAAACCGAAATGGGAGG | GTCTGCGAAAGCCGAGAT |
