# Supplementary material for: Genome-wide identification and structural analysis of the BMP gene family in Triplophysa dalaica
Source: BMC Genomics. 2024 Feb 19;25:194. doi: 10.1186/s12864-024-10049-z (PMC10875767; doi:10.1186/s12864-024-10049-z)
Supplement: Supplementary file 3 — Additional file 3: Table S3. NCBI accession numbers for the BMP protein sequences of the four species. [file 12864_2024_10049_MOESM3_ESM.docx]

**Table S3** NCBI accession numbers for the *BMP* protein sequences of the four species

| Species name | Gene name | NCBI accession number |
| --- | --- | --- |
| *Danio rerio* | *BMP1a* | AAI63535.1 |
| *Danio rerio* | *BMP1b* | BAE80513.1 |
| *Danio rerio* | *BMP2a* | AAI63036.1 |
| *Danio rerio* | *BMP2b* | AJF23294.1 |
| *Danio rerio* | *BMP3a* | NP_001071233.1 |
| *Danio rerio* | *BMP3b* | ACA57845.1 |
| *Danio rerio* | *BMP4* | AAC60285 |
| *Danio rerio* | *BMP5* | NP_957345 |
| *Danio rerio* | *BMP6* | AAI65212.1 |
| *Danio rerio* | *BMP7a* | NP_571396.1 |
| *Danio rerio* | *BMP7b* | NP_001070614.2 |
| *Danio rerio* | *BMP8a* | NP_001038436.1 |
| *Danio rerio* | *BMP9* | ACA57846.1 |
| *Danio rerio* | *BMP10a* | Gene ID: 571601 |
| *Danio rerio* | *BMP10b* | NP_001124072.1 |
| *Danio rerio* | *BMP11* | NP_998140.1 |
| *Danio rerio* | *BMP12* | XP_694563.2 |
| *Danio rerio* | *BMP13a* | NP_001153466.1 |
| *Danio rerio* | *BMP13b* | XP_694563.2 |
| *Danio rerio* | *BMP14* | XP_002662587.1 |
| *Danio rerio* | *BMP15* | NP_001018320.1 |
| *Danio rerio* | *BMP16* | NP_001165247.1 |
| *Xenopus laevis* | *BMP1* | P98070.1 |
| *Xenopus laevis* | *BMP2* | P25703.1 |
| *Xenopus laevis* | *BMP3a* | Q7T2X7.1 |
| *Xenopus laevis* | *BMP3b* | Q7T2X6.1 |
| *Xenopus laevis* | *BMP4* | P30885.1 |
| *Xenopus laevis* | *BMP5* | XP_004914553.1 |
| *Xenopus laevis* | *BMP6* | NP_001106378.1 |
| *Xenopus laevis* | *BMP7a* | NP_001080866.1 |
| *Xenopus laevis* | *BMP7b* | XP_017947617.1 |
| *Xenopus laevis* | *BMP8a* | XP_018105085.1 |
| *Xenopus laevis* | *BMP9* | XP_004915890.1 |
| *Xenopus laevis* | *BMP10* | XP_002935357.1 |
| *Xenopus laevis* | *BMP11* | XP_018102899.1 |
| *Xenopus laevis* | *BMP13* | XP_018123657.1 |
| *Xenopus laevis* | *BMP14* | NP_001086466.1 |
| *Xenopus laevis* | *BMP15* | XP_018085100.1 |
| *Cyprinus carpio* | *BMP1a-1* | LC219865 |
| *Cyprinus carpio* | *BMP1a-2* | LC219866 |
| *Cyprinus carpio* | *BMP1b-1* | LC219867 |
| *Cyprinus carpio* | *BMP1b-2* | LC219868 |
| *Cyprinus carpio* | *BMP2a-1* | LC219869 |
| *Cyprinus carpio* | *BMP2a-2* | LC219870 |
| *Cyprinus carpio* | *BMP2b-1* | LC219871 |
| *Cyprinus carpio* | *BMP2b-2* | LC219872 |
| *Cyprinus carpio* | *BMP3a-1* | LC219873 |
| *Cyprinus carpio* | *BMP3a-2* | LC219874 |
| *Cyprinus carpio* | *BMP3b-1* | LC219875 |
| *Cyprinus carpio* | *BMP3b-2* | LC219876 |
| *Cyprinus carpio* | *BMP4-1* | LC219877 |
| *Cyprinus carpio* | *BMP4-2* | LC219878 |
| *Cyprinus carpio* | *BMP5-1* | LC219879 |
| *Cyprinus carpio* | *BMP5-2* | LC219880 |
| *Cyprinus carpio* | *BMP6a-1* | LC219881 |
| *Cyprinus carpio* | *BMP6a-2* | LC219882 |
| *Cyprinus carpio* | *BMP7a-1* | LC219883 |
| *Cyprinus carpio* | *BMP7a-2* | LC219884 |
| *Cyprinus carpio* | *BMP7b-1* | LC219885 |
| *Cyprinus carpio* | *BMP7b-2* | LC219886 |
| *Cyprinus carpio* | *BMP8a-1* | LC219887 |
| *Cyprinus carpio* | *BMP8a-2* | LC221568 |
| *Cyprinus carpio* | *BMP9-1* | LC219888 |
| *Cyprinus carpio* | *BMP9-2* | LC219889 |
| *Cyprinus carpio* | *BMP10a-1* | LC221569 |
| *Cyprinus carpio* | *BMP10a-2* | LC221570 |
| *Cyprinus carpio* | *BMP10b-1* | LC219890 |
| *Cyprinus carpio* | *BMP10b-2* | LC219891 |
| *Cyprinus carpio* | *BMP11-1* | LC219892 |
| *Cyprinus carpio* | *BMP11-2* | LC219893 |
| *Cyprinus carpio* | *BMP12-1* | LC219894 |
| *Cyprinus carpio* | *BMP12-2* | LC219895 |
| *Cyprinus carpio* | *BMP13a-1* | LC219896 |
| *Cyprinus carpio* | *BMP13a-2* | LC219897 |
| *Cyprinus carpio* | *BMP13b-1* | LC219898 |
| *Cyprinus carpio* | *BMP13b-2* | LC219899 |
| *Cyprinus carpio* | *BMP14-1* | LC219900 |
| *Cyprinus carpio* | *BMP14-2* | LC219901 |
| *Cyprinus carpio* | *BMP15-1* | LC219861 |
| *Cyprinus carpio* | *BMP15-2* | LC219862 |
| *Cyprinus carpio* | *BMP16-1* | LC219863 |
| *Cyprinus carpio* | *BMP16-2* | LC219864 |
| *Homo sapiens* | *BMP1* | NP_001190.1 |
| *Homo sapiens* | *BMP2* | ACV32596.1 |
| *Homo sapiens* | *BMP3a* | P12645.2 |
| *Homo sapiens* | *BMP3b* | NP_004953.1 |
| Homo sapiens | *BMP4* | P12644.1 |
| Homo sapiens | *BMP5* | P22003.1 |
| Homo sapiens | *BMP6* | P22004.1 |
| Homo sapiens | *BMP7* | P18075.1 |
| Homo sapiens | *BMP8a* | Q7Z5Y6.2 |
| Homo sapiens | *BMP9* | Q9UK05.1 |
| Homo sapiens | *BMP10* | O95393.1 |
| Homo sapiens | *BMP11* | O95390.1 |
| Homo sapiens | *BMP12* | NP_878248.2 |
| Homo sapiens | *BMP13* | NP_001001557.1 |
| Homo sapiens | *BMP14* | P43026.4 |
| Homo sapiens | *BMP15* | O95972.2 |
